# Post-laparoscopic oral contraceptive combined with Chinese herbal mixture in treatment of infertility and pain associated with minimal or mild endometriosis: a randomized controlled trial

**DOI:** 10.1186/1472-6882-14-222

**Published:** 2014-07-05

**Authors:** Shaomi Zhu, Dong Liu, Wei Huang, Qiushi Wang, Qiuyi Wang, Lu Zhou, Guimei Feng

**Affiliations:** 1 Department of Obstetrics and Gynecology, West China Second University Hospital of Sichuan University, Renminnanlu 3 Duan 20hao, Chengdu, Sichuan 610041, China

**Keywords:** Endometriosis, Infertility, Oral contraceptive pills, Herbal medicine

## Abstract

**Background:**

Endometriosis affects fertility negatively. The study aims to evaluate whether laparoscopic surgery combined with oral contraceptive or herbs were more effective than laparoscopic alone in improving fecundity and pelvic pain in women with minimal/mild endometriosis.

**Methods:**

A randomized controlled trial (RCT) was conducted in 156 infertile women with minimal/mild endometriosis. After laparoscopic surgery, patients were randomized to three groups: in Group A (n = 52) oral contraceptive (OC) was administered one pill a day, continuous for 63 days without intervals, in Group B (n = 52) OC was administered as above and then Dan’e mixture was added 30 g/day for the latter 30 days, and in control Group C (n = 52) patients tried to get pregnant after surgery without complementary treatment. The follow-up periods were 12 months in Group C and 14 months in complementary medical treatment Group A and B. The pregnant women were further followed up, and labor and pregnancy outcomes were assessed. Primary outcome was pregnancy rate (PR) and live birth rate (LBR). Secondary outcomes included changes of pelvic pain visual analog scale scores and side effects. Analyses were done as intention-to-treat.

**Results:**

The PR was 46.80% (73/156), and the LBR was 69.86% (51/73). Of the 73 pregnancies, 60 occurred within 12 months of follow-up and 7 of the remaining 13 patients underwent assisted reproductive technology for >1 year. No significant difference was observed in PR and LBR among the three groups. Patients given medical treatment (OCs or OCs plus herbal medicine) had significantly decreased pain scores compared with the laparoscopy alone group.

**Conclusions:**

Combination of laparoscopy with OCs or OCs and herbal medicine does not have more advantages than laparoscopy alone in improving fertility of women with minimal/mild endometriosis.

**Trial registration:**

ChiCTR-TRC-11001820

## Background

Endometriosis is a condition in which endometrial glands and stromal implants grow outside the uterus, causing chronic pelvic pain and infertility. It is reported that 30–50% of women with endometriosis are infertile [1]. Of the patients with endometriosis, 32% have moderate to severe disease, while 58% have minimal or mild endometriosis [2]. The pathogenesis of mild/minimal endometriosis associated with infertility is rather unclear. Therefore, current therapy for these women involves the use of surgical methods including laparoscopy, expectant treatment, induced ovulation, and assisted reproductive technology (ART) [3,4] and medication that induces a pseudomenopause or pseudopregnancy state.

A Canadian RCT involving 341 women with minimal or mild disease followed up for 36 weeks after laparoscopy reported that surgery was more effective than no treatment and treatment with pharmaceutical agents alone [5]. However, with laparoscopy, all parts of the lesion cannot be removed completely. Moreover, postoperative treatment with drugs is still a controversial issue [6-8]. Therefore, there is a need to determine the usefulness of postoperative medical treatment in these cases, which is what our study set out to do.

As one of the first-line treatments for pelvic pain associated with endometriosis, OCs are highly safe, fewer side effects, and cost-effective. However, only a few RCTs have reported the use of OCs in the treatment of minimal/mild endometriosis after surgery [9,10]. Therefore, the ability of laparoscopy combined with OCs to improve fertility that occur secondary to minimal/mild endometriosis remains debatable.

Chinese traditional medicines have also long been in use for the treatment of endometriosis. Dan’e mixture is a combination of qi-regulating and blood-vitalizing herbs, mainly composed of danshen (salvia), ezhu (zedoaria), chishao (red peony), danggui (tang-kuei), chaihu (bupleurum) and yanhusuo (corydalis). Dan’e mixture displayed similar effects to Danazol in the treatment of infertility and pelvic pain associated with endometriosis. However, no clinical data on Dan’e mixture for treatment in infertile women with minimal/mild endometriosis is found now. A rigorous randomized controlled trial is necessary. In our study, we speculated on whether a combination of OCs and herbal medicines can improve fertility in these women. There is currently no published study comparing OCs with a combination of OCs and herbs. In addition, there is no adequate powered randomized trial that has studied the effectiveness of laparoscopy combined with medical treatment compared to surgery alone in women with infertility and pain secondary to minimal/mild endometriosis.

In this prospective, randomized controlled study, we compared laparoscopy alone with laparoscopy followed by treatment with OCs or a combination of OCs and the Dan’e mixture in the treatment of minimal/mild endometriosis, primarily with regard to improvement of fecundity and alleviation of pelvic pain.

## Methods

### Study population

The study was conducted at the Department of Obstetrics and Gynecology, West China Second Hospital of Sichuan University, from February 2011 to May 2013. It was conducted in accordance with the ethical principles of the Helsinki Declaration, and the study protocol was approved by the ethics committee of the hospital. The trial is reported and was analyzed in accordance with the CONSORT criteria. All the included patients signed an informed consent form before participation. The study population was infertile women with minimal or mild endometriosis confirmed by laparoscopy, according to the revised American Fertility Society (r-AFS) classification (r-AFS score < 16) [11]. The study included women aged 20 to 40 years who wished to conceive and had failed to get pregnant after at least 12 months of unprotected intercourse. Women were excluded if they had previously undergone medical or surgical treatments for endometriosis; if their infertility resulted from problems with the ovary, fallopian tube, or uterus, or other causes such as adenomyosis, ovarian endometrioma or deep endometriosis; or if the male partner had abnormal sperm cells or was suspected to have any gynecologic malignancies. Women with contraindications for OCs such as severe diabetes and hypertension, hepatic or renal dysfunction, and idiopathic vagina bleeding were also not included.

### Sample size

Sample size calculations were based on the assumption that there would be a 15% difference between pregnant rate [5]. When an α value of 0.05 with power 80% and a 20% dropout rate were considered, the sample size of each group was determined to be 52.

### Study design

**Figure 1:**
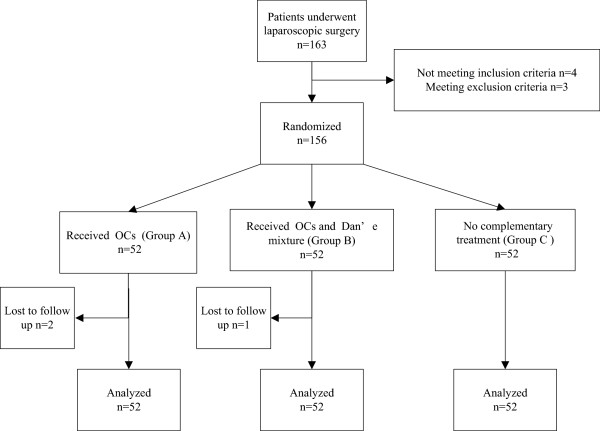
Flow chart of study.

All patients underwent laparoscopy under general anesthesia. All apparent endometriosis lesions, including superficial endometriomas and implant lesions, were excised or cauterized by monopolar or bipolar electrocauterization. The pelvic and fallopian adhesions were detected and lysed to restore normal anatomy. The patency of the fallopian tube was examined using methylene blue during laparoscopy, and finally about 2000 ml of 0.9% normal saline was used to wash the pelvis thoroughly.After the operation, the patients were randomly allocated to three groups: Group A: an OC (Marvelon: 30 μg ethinyl estradiol and 150 μg desogestrel/tablet) was administered one tablet continuously for 63 days, Group B: the OC was administered one tablet continuously for 63 days and the Dan’e mixture (manufactured by DIHON Medicine, Yunnan Province, China) was administered at 30 g/day for the latter 30 days, Group C: no medical treatment was given (Figure 1). The patients in Group C were prepared to conceive after their one-month visit, and the patients in Group A and Group B were prepared to conceive after they experienced withdrawal bleeding at the end of medical treatment. Each participant was asked to self-estimate pelvic pain (dysmenorrhea, noncyclic pelvic pain, and deep dyspareunia) using a visual analogue scale (VAS), with a score of 0 indicating no pain and 10 indicating the worst pain imaginable. The random allocation was conducted using a computer-generated list of random numbers. The codes A, B, and C were placed separately in three sealed envelopes; they were sequentially numbered and then chronologically opened in the ward only after an eligible patient was identified. The study would be cancelled when the participant appeared apparently side effect of any medicine or desired to stop the treatment.

### Follow-up

All participants completed their one-month visit after surgery, where their menstrual status was noted and their recovery was ensured. Then, they were regularly followed up via the phone or outpatient visits every three months for 12 months in Group C and 14 months in complementary medical treatment Group A and B. The pregnant women were further followed up, and labor and pregnancy outcomes were assessed. Those who failed to conceive at 12 months of follow-up were advised to undergo ART in the form of intrauterine insemination (IUI) or in vitro fertilization (IVF). The VAS pain score and side effects of the medical treatment such as gastrointestinal reactions, irregular vaginal bleeding, and weight gain were assessed at every visit.

The primary outcomes were PR and live birth (delivery of a take-home baby) rate (LBR). Miscarriages (fetal loss at <20 weeks of gestation, preterm delivery (births between 20 to 37 weeks), stillbirth (fetal loss after ≥28 weeks of gestation or birth weight ≥ 1000 g) and obstetric complications were also defined. The secondary outcomes were VAS pain scores, and the side effects of the medicine were also investigated.

### Statistical analysis

Statistical analyses were performed with SPSS 17.0 for Windows. Continuous data were tested for normality using histograms and normal Q-Q plots. For variables with abnormal distribution, the non-parametric Kruskal-Wallis test and Wilcoxon’s signed-rank test were used to examine intra-group and inter-group differences in VAS scores. Dunn's post test was used for pairwise comparison following Kruskal-Wallis test. For qualitative variables with normal distribution, the χ^2^ test or Fisher’s exact test was used for count data, as appropriate according to sample size, and independent-samples *t*-test or one-way ANOVA was used for parametric data. Statistical significance was accepted at P < .05. All analyses were two-tailed and conducted on intention-to-treat basis according to CONSORT criteria. Dropouts were categorized based on their status at last contact.

## Results

Flow of study participants is shown in Figure 1. A total of 163 women with minimal or mild endometriosis were enrolled, out of which 7 dropped out: 4 delayed their pregnancy plan temporarily, and 3 had contraindications for OCs or had undergone previous medical treatment for endometriosis. The remaining 156 were randomized into Group A (n = 52; OC only), Group B (n = 52; OC + Dan’e mixture) and Group C (n = 52; no medical treatment). The mean duration of follow-up was 22.17 ± 3.39 months (range, 14–27 months); three participants were lost to follow-up (two in Group A and one in Group B) because their telephone numbers had changed and they could not be contacted. None of the participants withdrew from the trial due to side effects. Therefore, 156 cases were finally included in the intention-to-treat analysis, and the worst-case scenario was applied in the analysis for the three lost to follow-up. Baseline characteristics of the intention-to-treat population are shown in Table 1. The three groups were comparable with regard to their demographic and clinical characteristics (P > .05).

**Table 1:** Baseline characteristics of the 156 endometriosis patients

	**Group A (n = 52)**	**Group B (n = 52)**	**Group C (n = 52)**
Age (years)	28.1 ± 2.2	28.6 ± 5.1	29.44 ± 4.8
Duration of infertility (years)	2.5 ± 1.9	2.7 ± 1.8	2.6 ± 1.6
BMI (kg/m^2^)	19.8 ± 3.1	20.3 ± 3.1	20.5 ± 2.4
r-AFS stage (n, %)			
Stage I	34 (65.38)	30 (57.69)	28 (53.85)
Stage II	18 (34.62)	22 (42.31)	24 (46.15)
VAS pain score	38.5 (32–47)	35 (22–58)	28 (23.5–44.5)

Data are expressed as mean ± standard deviation and median (interquartile range) or as number and percentage (%).

A total of 73 pregnancies occurred during nearly two years of follow-up and the PR was 46.80% (73/156). Among them, 60 pregnancies occurred within the 12 months after completion of treatment. Among the remaining 13 pregnancies that followed, 4 conceived without any additional treatment, 3 were given medication for induction of ovulation, and 7 underwent ART (IUI, 2; IVF-ET, 5). Up to now, there have been 51 live births, including cases of 7 preterm labor (LBR, 69.86%; 51/73); among the remaining pregnancies, miscarriages occurred in 14 cases, stillbirth in 1 case, and ectopic pregnancies in 3 cases, and the gestational period was longer than 28 weeks in 4 cases.

The pregnancy rate within 12 months of follow-up was 38.46% (60/156): 20 in Group A (38.46%, 20/52), 16 in Group B (30.77%, 16/52), and 24 in Group C (46.15%, 24/52). There were three cases of ectopic pregnancy (one in Group A and two in Group C), ten spontaneous abortions (four in Group A, three in Group B and three in Group C), and one stillbirth in Group A; therefore, there were 46 live births (76.67%, 46/60). No significant difference in PR and LBR was observed between the three groups (P > .05) (Table 2).

**Table 2:** Intention-to-treat analysis of pregnancy and births according to treatment allocation within 12 months of follow-up

	**Group A (n = 52)**	**Group B (n = 52)**	**Group C (n = 52)**
Pregnancy n (%)	20 (38.46)	16 (30.77)	24 (46.15)
Outcome of pregnancy n (%)			
Live birth	14 (70.00)	13 (81.25)	19 (79.16)
Miscarriage (<28 weeks)	4 (20.00))	3 (18.75)	3 (12.50)
Stillbirth (>28 weeks)	1 (5.00)	0	0
Ectopic pregnancy	1 (5.00)	0	2 (8.33)

Assumption for intention-to-treat analysis: those lost to follow-up did not conceive.

Data were expressed as number and percentage (%).

The symptoms of pelvic pain improved and VAS scores decreased in the three groups at the sixth month of follow-up compared to the baseline (P < .05). Patients treated with either OC or OC plus Dan’e mixture had significantly lower scores than those who received no medical treatment (P < .05). No significant difference in pain scores was observed between the two medical treatments (P > .05) (Table 3).

**Table 3:** VAS scores over the six-month study period

	**Group A (n = 52)**	**Group B (n = 52)**	**Group C (n = 52)**
Baseline	38.5 (0–63)	35 (0–82)	28 (0–61)
Six months after treatment	15 (0–46)	19 (0–52)	29 (0–56)

Values of pain score are expressed as median (interquartile range).

The potentially treatment-related adverse events experienced irregular vaginal bleeding and breast tenderness. No significant difference was observed in the incidence of these adverse events between the two groups (Table 4).

**Table 4:** Side effects in the medical treatment groups

	**Group A (n, %)**	**Group B (n, %)**
Irregular vaginal bleeding	14 (26.92)	18 (34.62)
Breast tenderness	13 (25.00)	10 (19.23)
Weight gain	9 (17.31)	6 (11.54)
Gastrointestinal discomfort	4 (7.69)	6 (11.54)

Data are expressed as number and percentage (%) and were compared using the χ2 test or Fisher’s exact.

## Discussion

In this prospective, randomized, controlled study, our main purpose was to evaluate the therapeutic effectiveness of laparoscopy combined with different medicine regimens in infertile women with minimal or mild endometriosis. Although a lot of studies have demonstrated no difference in pregnancy rates with preceding ovulation suppression with oral contraceptives, progestins, or danazol in infertile women with endometriosis, only a few trials have investigated whether laparoscopy combined with medical treatment is beneficial for the treatment of women with minimal/mild endometriosis [12].

The spontaneous pregnancy rate within 12 months was 38.46%, but it is lower than the rate expected in infertile women after IVF/ICSI treatment (64.6%) [13]. This indicates that endometriosis may not be the sole factor causing infertility in these women, and therefore destruction of visible endometriotic implants and adhesiolysis may not be sufficient to combat infertility.

As we know, postoperative complementary medication treatments with minimal/mild endometriosis in women who wish to conceive is still a controversial issue. In this study, we chose a continuous 63-day OC administer regimen, in order to avoid withdrawal bleeding and effectively suppress ectopic foci [14,15]. Unfortunately, we did not find this regimen to have any advantages with regard to enhancing fertility in these women. Medical suppression of ovulation delays conception only until the treatment is completed, and it may in fact be counterproductive in the case of infertile patients. This drawback becomes even more pronounced in infertile patients with surgically treated endometriosis, because traditional post-operative hormone therapy interferes with ovulation during a critical period for conception [16].

It was reported that the traditional medicine can improve women’s fecundity. As we know, the traditional medicine usually need longer time to work, the time for conception after surgery is confined. Therefore, a combination of OC and traditional medicine is designed in the study to increase the effects of herbs in a short time. However, we failed to convince improvement of herbs for women’s fecundity. Both regimen OC alone and combination of OC and herbs were not more effective at enhancing fertility compared with surgery alone. One of drawback in this study is no traditional medicine alone regimen. We need to conduct a future study in which the traditional medicine alone be designed to determine its role for improve female fecundity.

When individual pain scores were assessed at 6 months, there was a reduction in pain scores in all patients, independent of the group to which they were randomized. This is similar to previously reported rates of response in women with minimal and mild endometriosis [17]. However, we found that the pain scores in the two medical treatment groups were significantly lower than those in the control group at the 8th and 6th month after surgical treatment. This is in agreement with the results of present and past studies, in which the benefits of OCs in managing symptoms associated with endometriosis have been clearly demonstrated [13,14]. Interestingly, no difference in pain scores was identified between the two medical treatment groups.

Miscarriage occurred in approximately 19.30% of the cases in this study, which suggests an increased incidence in spontaneous abortion and decreased LBR in women with endometriosis. Similar results were also observed in a multicentric Italian study [18]. The definite mechanism by which endometriosis may affect spontaneous pregnancy loss is as yet undetermined. A putative luteal-phase defect associated with endometriosis may be the problem [19]. However, the pathogenesis of the luteal-phase defect is unclear; it is believed to be caused by a proliferative phase defect, low expression of intergrin and HOX 10, progesterone resistance, or abnormality in the junctional zone, which result in increased rate of fetal loss in women with endometriosis [20,21]. The pathogenesis of pregnancy loss resulting from endometriosis needs to be studied further.

A major strength of our study is the rigorous randomized, prospective and the sufficient sample size. The main drawback is the absence of blinding to patients and the pilot nature of the study. It was not possible to blind participants to treatment allocation since the treatment involved the patients themselves taking medication at home and the control group received no intervention. Therefore, the present study was not a double-blind study, and consequently, there might be a bias in favor of the treatment group.

## Conclusions

In conclusion, there is no evidence from this study that combining surgery with medication with OCs or OCs and herbs had better benefits in terms of improving fertility than laparoscopy alone; therefore, we think that in cases of minimal/mild endometriosis, it is better to conceive immediately after surgery, as there is a 30% chance of conceiving during the 6–12 month period following laparoscopy. Those who fail to conceive should be treated with superovulation/IUI or IVF according to their preferred choice, the woman’s age and other factors. Moreover, laparoscopy is effective for relieving pain associated with minimal/mild endometriosis, but there is no evidence that medicine combination of OCs and herbal mixtures have these pain relieving effects better than OCs alone.

## Competing interests

The authors declare that they have no competing interests.

## Authors’ contributions

SMZ and DL were responsible for protocol development, study implementation, data analysis. SMZ drafting of manuscript and report-writing. WH was responsible for the whole study and critically reviewed manuscript. QSW and QYW were part of research team and contributed to study implementation and analysis of qualitative data. LZ and GMF was responsible for quantitative data entry, data cleaning and analysis. All authors read and approved the final manuscript.

## Pre-publication history

The pre-publication history for this paper can be accessed here:

http://www.biomedcentral.com/1472-6882/14/222/prepub
